# Remdesivir therapy associated with Bradycardia in SARS‐CoV2

**DOI:** 10.1002/clc.23700

**Published:** 2021-07-28

**Authors:** Sabina Kumar, Christina Arcuri, Sumanta Chaudhuri, Rahul Gupta, Mahendra Aseri, Pranav Barve, Shivang Shah

**Affiliations:** ^1^ Department of Internal Medicine Hemet Global Medical Center Hemet California USA; ^2^ Division of Cardiology Loma Linda University School of Medicine Loma Linda California USA; ^3^ Division of Cardiology University of California Riverside School of Medicine Riverside California USA

Thank you Barkas et al. for your interest in our article titled “A novel study on SARS‐COV‐2 virus associated bradycardia as a predictor of mortality‐retrospective multicenter analysis.”^1^ In our large multicenter retrospective study, 28.7% of patients who received remdesivir developed an absolute bradycardic (HR <50) response during their hospitalization. Unfortunately, we could not comment if remdesivir was the sole cause of bradycardia because there was a high incidence of bradycardia in individuals who were not on remdesivir.

The possible mechanism for remdesivir causing bradycardia is still up for debate. The active metabolite is a nucleotide triphosphate which is a derivate similar to adenosine triphosphate (ATP).^2^ ATP has been shown to induce SA nodal automaticity through vagal simulation.^3^ In addition, ATP metabolite adenosine exerts negative chrontropic and dromotropic effects, which could affect AV nodal conduction.^3^

In the future, we agree with Barkas et al. that remdesivir and development of bradycardia needs to be investigated further. Touafchia et al. used the WHO safety report database reported a 31% incidence of bradycardia in those individuals receiving remdesivir. 75% of patients with bradycardia went on to develop serious complications including death.^4^ These results were very similar as seen in our study as well.

Barkas et al. bring up a good point on the relationship between onset of remdesivir exposure and development of bradycardia. Remdesivir has a high affinity to bind to viral polymerases; however, there is a chance for cross‐reactivity with human mitochondrial RNA polymerase, which could lead to mitochonridal dysfunction and subsequent cardiomyocte toxicity. Choi et al. showed that the cytotoxic effects of remdesivir increased overtime (48 hours vs. 24 hours), in addition to reducing cell viability.^5^

Multiple case reports have also discussed remdesivir‐causing bradycardia.^5^ Gubitosa et al. wrote a case report on a 54 y/o female who had marked sinus bradycardia (HR ~ 38 BPM) within 24 hours of receiving remdesivir.^6^ Sanchez‐Codez and colleagues described a case report where a 13 year old male had a heart rate around 40 BPM after his third dose of remdesivir.^7^ Fralick et al. and team recently reported a patient who developed bradycardia (HR ~50 BPM) 48 hours after the administration.^8^ Through our literature review, we found more than 10 case reports discussing remdesivir administration and subsequent bradycardia. In most of the case reports, there was only a transient bradycardic event followed by normalization of heart rate after the discontinuation of the medication.

We are in the process of pursuing a retrospective analysis looking specifically at the timing of remdesivir and subsequent bradycardia. We invite anyone to send a message to the corresponding author if they want to collaborate on our study.

## CONFLICT OF INTEREST

The author(s) declare no conflict of interest for this work.
